# Diabetes and SARS-CoV-2–Is There a Mutual Connection?

**DOI:** 10.3389/fcell.2022.913305

**Published:** 2022-06-13

**Authors:** Anna P. Jedrzejak, Edyta K. Urbaniak, Jadwiga A. Wasko, Natalia Ziojla, Malgorzata Borowiak

**Affiliations:** ^1^ Institute of Molecular Biology and Biotechnology, Adam Mickiewicz University, Poznan, Poland; ^2^ Center for Cell and Gene Therapy, Stem Cell and Regenerative Medicine Center, Baylor College of Medicine, Texas Children’s Hospital, Methodist Hospital, Houston, TX, United States; ^3^ McNair Medical Institute, Baylor College of Medicine, Houston, TX, United States

**Keywords:** diabetes, SARS-CoV-2, pancreas, infection, receptor, β-cells (beta-cells)

## Abstract

SARS-CoV-2, a newly emerged virus described for the first time in late 2019, affects multiple organs in humans, including the pancreas. Here, we present the bilateral link between the pathophysiology of diabetes and COVID-19, with diabetes being COVID-19 comorbidity, and a complication of SARS-CoV-2 infection. Analysis of clinical data indicates that patients with chronic conditions like diabetes are at increased risk of severe COVID-19, hospitalization, ICU admission, and death compared to the healthy subjects. Further, we show that SARS-CoV-2 infection might be also associated with the development of new-onset diabetes and diabetic ketoacidosis. We then discuss the options for studying SARS-CoV-2 infection in pancreatic settings, including the use of human pluripotent stem cell-derived pancreatic organoids. Further, we review the presence of SARS-CoV-2 receptors in different pancreatic cell types and the infection efficiency based on pancreatic sections from COVID-19 patients and primary human islet *in vitro* studies. Finally, we discuss the impact of SARS-CoV-2 infection on human pancreatic cell homeostasis, focusing on β-cells.

## Graphical Abstract

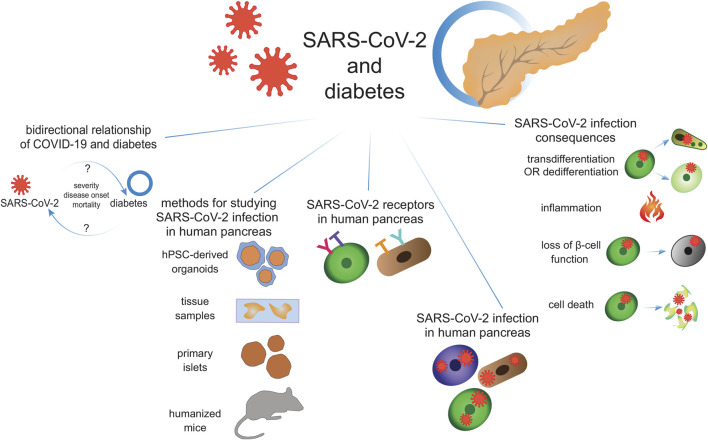

## Introduction

In December 2019, in Wuhan, the Hubei province of central China, a new viral strain causing severe pneumonia was first reported and quickly escalated into a pandemic that paralyzed the world’s daily functioning in terms of economy, healthcare, cultural events, education, and tourism. Intensive worldwide efforts led to the identification of the pathogen as a close relative of the viruses that cause Severe Acute Respiratory Syndrome (SARS) and Middle East Respiratory Syndrome (MERS), belonging to the coronavirus family and causing lethal respiratory infections in humans (Lu et al., 2015; Vijay and Perlman, 2016; Gordon et al., 2020). By similarity, the newly emerged virus was named SARS-CoV-2, and the resultant disease coronavirus disease 2019 (COVID-19).

So far, five SARS-CoV-2 variants of concern have been identified, differing in terms of the symptoms, incubation periods, contagiousness, and the severity of COVID-19. The course of the disease varies from asymptomatic, through mild flu-like symptoms, up to severe pneumonia and respiratory failure, resulting in hospitalization and/or intensive care unit (ICU) admission, with initially high fatality rates. Most common symptoms include fatigue, muscle and joint pain, headache, cough, fever, and breathing difficulties. Additionally, conjunctivitis and loss of smell or taste can occur together with COVID-19. Further, the δ variant, ​​a highly contagious SARS-CoV-2 virus variant that was first identified in India, may cause symptoms similar to sinus infection and gastrointestinal problems, including nausea, vomiting, and diarrhea. Data suggest that omicron (Ο), the most recent variant, while very contagious, is generally less severe (Stokes et al., 2020; Cascella et al., 2022).

Moreover, cases of so-called “long COVID-19” are reported, not only in individuals with severe COVID-19 but also in those in which the infection was mild or moderate. Symptoms of long COVID-19 include fatigue, shortness of breath, chest pain, joint pain, palpitations, continued dysfunctions of taste and smell, hair loss, headaches, memory and attention deficits, and psychological disorders. People with long COVID-19 experience these symptoms weeks or even months after the initial infection, most probably as a consequence of tissue damage (Ayoubkhani et al., 2021; Davis et al., 2021; Ziauddeen et al., 2022).

With the high incidence of COVID-19, it quickly turned out that certain medical conditions negatively influence the COVID-19 severity, and increase hospitalization and death rates (Raghavan et al., 2020; Williamson et al., 2020; Xu et al., 2020; Deng et al., 2021). Moreover, SARS-CoV-2 negatively affects multiple organs, often leaving recovered patients with cardiological, neurological, and metabolic complications, with diabetes mellitus suggested as one of them.

Diabetes is a chronic metabolic disease with a prevalence of almost 422 million people affected, as reported in 2016, and with a projected increase to 700 million by 2045 (WHO, 2020). Diabetes is defined by the loss of blood glucose homeostasis, due to the inadequate action of insulin, a hormone produced by the endocrine β-cells of the pancreas. Glucose homeostasis is disturbed as a result of either autoimmune-mediated destruction of β-cells (as in type 1 diabetes, T1D) or insufficient β-cell mass, often coupled with peripheral insulin resistance (as in type 2 diabetes, T2D). So far, the golden-standard treatments include insulin-sensitizing drugs for T2D, such as metformin, or external insulin delivery, combined with regular blood tests.

In this review, we focus on the bidirectional relationship between COVID-19 and diabetes, with diabetes being 1) comorbidity that increases COVID-19 severity and mortality, and 2) a complication of SARS-CoV-2 infection. We discuss the outcomes and the death rates of COVID-19 patients with diabetes in comparison to the healthy population. We also highlight the case reports showing diabetes emerging due to SARS-CoV-2 infection in previously non-diabetic adults and children. We then discuss the different options for studying SARS-CoV-2 infection in human pancreas, including autopsy samples, primary islets, pluripotent stem cell-derived three-dimensional (3D) pancreatic organoids, and humanized mice. Next, we extensively review the current state of knowledge concerning the SARS-CoV-2 entrance into the pancreatic cells, including the presence and expression level of SARS-CoV-2 receptors in pancreatic cell types, and the presence of viral proteins. We show that pancreatic cell permissiveness for SARS-CoV-2 infection is highly variable, possibly reflecting the natural variability in the receptor expression level. Finally, we discuss the possible mechanisms of pancreatic cell deterioration through the course of COVID-19, focusing on β-cell survival and function.

## Can Diabetes Increase the Risk of SARS-CoV-2 Infection?

Diabetes often leads to multiple complications, including neuropathy, nephropathy, and retinopathy, shortening life expectancy by an average of 10 years (Astrup and Finer, 2000). Long-term hyperglycemia commonly occurs in diabetes, often leading to the dysfunction of the immune response, particularly at the level of phagocytosis and chemotaxis, along with significantly reduced cytokine secretion (Alexandraki et al., 2008; Erener, 2020). Changes in cellular immunity in people with diabetes are associated with a higher susceptibility to bacterial, fungal, and yeast infections (Hine et al., 2017; Carey et al., 2018; Kim et al., 2019). As for viruses, the higher infection risk in diabetic patients remains elusive, with different reports showing either small correlation or no correlation (Shah and Hux, 2003; Hine et al., 2017). So far there is no strong evidence suggesting a higher risk of SARS-CoV-2 infection in diabetes patients, as the number of patients with diabetes within COVID-19 cases is comparable to the prevalence of diabetes within the general population.

Of note, T2D patients were shown to have significantly lower expression of the canonical SARS-CoV-2 receptor, angiotensin converting enzyme 2 (ACE2) (Reich et al., 2008; Chen et al., 2020) and they are often treated with ACE inhibitors and angiotensin II type-I receptor blockers, which upregulate ACE2 expression for its protective effects on the pancreas (Carlsson et al., 1998; Batlle et al., 2010). The increased ACE2 expression could hypothetically accelerate SARS-CoV-2 entrance into the cell and increase the viral load accumulated before immune system reaction, therefore making the infection more severe.

Further, high levels of the cell surface protein dipeptidyl peptidase-4 (DPP4) (Gao et al., 2022) have a protective effect against viral infections. People with diabetes have often decreased DPP4 expression which consequently might lead to increased susceptibility to SARS-CoV-2 infections (Raha et al., 2020).

## Can Diabetes Increase the Severity of COVID-19?

It has been shown that people with diabetes have increased severity and higher mortality rates in cases of influenza, viral pneumonia, and infection by enterovirus Coxsackie B (Muniyappa and Gubbi, 2020; de Jong et al., 2021; Müller et al., 2021; Samies et al., 2021). Further, worse viral infection outcome in diabetic patients was already observed during previous SARS-CoV and MERS-CoV epidemics (Yang et al., 2006; Hui et al., 2018). This suggests that the same effect might also be observed during SARS-CoV-2 infection.

In Table 1, we present the summary of the reports on the COVID-19 outcome in people with diabetes. The results of the multiple studies indicate that diabetes is associated with increased COVID-19 severity, higher rates of hospitalization and ICU admission as well as increased mortality in comparison to healthy subjects. Besides diabetes, the highest rate of ICU admission and mortality was shown for hypertension, which is frequently associated with diabetes, and pneumonia. ICU admission rate of diabetic patients was from 1.1- to 24-fold higher, with a mean 3.5-fold increase compared to people without diabetes. Two studies were exceptions where the ICU admission rate was lower for diabetic people (Charlotte et al., 2020; Huang C. et al., 2020), likely due to the small sample size. Mortality rates were 1.2- to 23-fold higher, with a mean 4.2-fold increase in diabetic patients, also with one exception (Al-Salameh et al., 2021). Moreover, people with diabetes are also on average 1.3-fold more likely to be hospitalized, than non-diabetic patients.

**TABLE 1:** COVID-19 outcome in people with diabetes and control population. Results for diabetic patients were compared to either people without any diagnosed comorbidities (“healthy”) or people without diabetes, but with unknown status of other comorbidities (“no diabetes”). Articles containing 1) specified number of patients examined in the particular study, and 2) a control group (either healthy patients with no comorbidities or non-diabetic patients that could have other comorbidities) allowing a direct comparison with diabetic patients, were included in the table. ICU–intensive care unit; T2D–type 2 diabetes;—no data; * the patients did not show any of the examined comorbidities; ** patients did not have diabetes but might have other comorbidities.

Source	Total Cases SARS-CoV-2 (+)	Health Status	SARS-CoV-2 (+); Not Hospitalized	SARS-CoV-2 (+), Hospitalized, Not Admitted to ICU	SARS-CoV-2 (+), Hospitalized, Admitted to ICU	Mortality
Italy, Istituto Superiore di Sanità, (2021)	7,910 (deceased patients)	healthy*: 230	–	–	–	230/7,910 (2.9%)
		T2D: 2 317	–	–	–	2 317/7,910 (29.3%)
England, Open-SAFELY, Williamson et al. (2020)	–	no diabetes**: 15,559 826	–	–	–	COVID-19-related: 6 837/15,559 826 (0.04%)
		diabetes: 1 718 566	–	–	–	COVID-19-related: 4 089/1 718 566 (0.24%)
China, Deng et al. (2020)	44,672	no diabetes**: 43,570	–	–	–	943/43,570 (2.2%)
		diabetes: 1 102	–	–	–	80/1 102 (7.3%)
United Kingdom, Barron et al. (2020)	–	no diabetes**: 58,244 220	–	–	–	COVID-19-related: 15,831/58,244 220 (0.03%)
		diabetes: 3 170 250	–	–	–	COVID-19-related: 7,867/3 170 250 (0.3%)
United States, Bode et al. (2020)	570	no diabetes**: 386	–	–	–	24/386 (6.2%)
		diabetes: 184	–	–	–	53/184 (28.8%)
China, Zhou et al. (2020)	191	healthy*: 100	–	–	–	18/100 (18%)
		diabetes: 36	–	–	–	17/36 (47.2%)
China, Guo et al. (2020)	174	no diabetes**: 137	–	–	–	5/174 (3.6%)
		diabetes: 37	–	–	–	4/37 (10.8%)
China, Xu et al. (2020)	703	healthy*: 502	–	–	–	8/502 (1.6%)
		diabetes: 64	–	–	–	12/64 (18.8%)
China, Li B. et al. (2020)	93	healthy*: 61	–	–	–	9/61 (14.8%)
		diabetes: 11	–	–	–	5/11 (45.5%)
Iran, Nikpouraghdam et al. (2020)	2 964	healthy*: 2 641	–	–	–	201/2 641 (7.6%)
		diabetes: 113	–	–	–	11/113 (9.7%)
United States, Chilimuri et al. (2020)	375	healthy*: 88	–	–	–	18/88 (20.5%)
		diabetes: 175	–	–	–	90/175 (51.4%)
United Kingdom, Adderley et al. (2022)	1 040	no diabetes**: 670	–	–	–	175/670 (26.1%)
		diabetes: 370	–	–	–	113 (30.5%)
Egipt, AbdelGhaffar et al. (2022)	3 712	no diabetes**: 2 557	–	–	–	562/2 557 (22%)
		diabetes: 1 155	–	–	–	338/1 155 (29.3%)
Italy, Ciardullo et al. (2021)	373	non-diabetes: 304	–	–	–	109/304 (35.9%)
		diabetes: 69	–	–	–	33/69 (47.8%)
Iran, Borzouei et al. (2021)	1 680	no diabetes**: 1 260	–	–	–	202/1 260 (16.0%)
		diabetes: 420	–	–	–	85/420 (20.2%)
Mexico, Ñamendys-Silva et al. (2021)	184	no diabetes**: 111	–	–	–	48/111 (43.2%)
		diabetes: 53	–	–	–	37/53 (69.8%)
Kuwait, Al-Ozairi et al. (2021)	5 089	no diabetes**: 3 903	–	–	–	113/3 903 (2.9%)
		diabetes: 1 186	–	–	–	131/1 186 (11.0%)
China, Yan et al. (2020)	193	no diabetes**: 145	–	–	60/145 (41.4%)	69/145 (47.6%)
		diabetes: 48	–	–	32/48 (66.7%)	39/48 (81.3%)
United States, Chow et al. (2020)	7,162	healthy*: 4 470	3 755/4 470 (84%)	305/4 470 (6.8%)	99/4 470 (2.2%)	–
		diabetes: 784	331/784 (42.2%)	251/784 (32%)	148/784 (18.9%)	–
China, Huang C. et al. (2020)	41	healthy*: 28	–	20/28 (71.4%)	8/28 (28.6%)	–
		diabetes: 8	–	7/8 (87.5%)	1/8 (12.5%)	–
China, Wang D. et al. (2020)	138	healthy*: 74	–	64/74 (86.5%)	10/74 (13.5%)	–
		diabetes: 14	–	6/14 (42.9%)	8/14 (57.1%)	–
United States , Argenzian et al. (2020)	1 000	no diabetes**: 628	–	382/628 (60.8%)	135/628 (21.5%)	–
		diabetes: 372	–	232/372 (62.4%)	101/372 (27.2%)	–
Switzerland, Charlotte et al. (2020)	196	healthy*: 34	–	25/34 (73.5%)	9/34 (26.5%)	–
		diabetes: 52	–	41/52 (78.8%)	11/52 (21.2%)	–
Iran, Tabrizi et al. (2021)	268	no diabetes: 141	–	–	16/141 (11.3%)	8/141 (5.7%)
		diabetes: 127	–	–	60/127 (47.2%)	22/127 (17.3%)
India, Raghavan et al. (2020)	845	no diabetes: 422	–	–	51/422 (12.1%)	25/422 (5.9%)
		diabetes: 423	–	–	80/423 (18.9%)	43/423 (10.2%)
China, Guan et al. (2020)	1 099	healthy*: 261	non-severe:194/261 (74.3%)	severe: 67/261 (25.7%)	–	–
		diabetes: 81	non-severe: 53/81 (65.4%)	severe: 28/81 (34.6%)	–	–
China, Zhang et al. (2021)	221	healthy*: 143	non-severe: 128/143 (89.5%)	severe: 15/143 (10.5%)	–	–
		diabetes: 22	non-severe: 15/22 (68.2%)	severe: 7/22 (31.8%)	–	–
China, Zhang et al. (2020)	788	healthy*: 528	common: 497/528 (94.1%)	severe: 30/528 (5.7%)	critical: 1/528 (0.2%)	–
		diabetes: 54	common: 42/54 (77.8%)	severe: 8/54 (14.8%)	critical: 4/54 (7.4%)	–
France, Sutter et al. (2021)	1 206	no diabetes: 603	–	–	118/603 (19.6)	91/603 (15.1%)
		diabetes: 603	–	–	126/603 (20.9)	111/603 (18.4)
Mexico, Peña et al. (2021)	323,671	no diabetes: 265,765	184,611/265,765 (69.5%)	81,154/265,765 (30.5%)		33,222/265,765 (12.5%)
		diabetes: 57,906	17,835/57,906 (30.8%)	40,071/57,906 (69.2%)		21,095/57,906 (36.4%)
China, Shang et al. (2021)	584	no diabetes: 500	–	–	29/500 (5.8%)	40/500 (8.0%)
		diabetes: 84	–	–	9/84 (10.7%)	17/84 (20.2%)
France, Al-Salameh et al. (2021)	432	no diabetes: 317	–	–	73/317 (23.0%)	68/317 (21.5%)
		diabetes: 115	–	–	40/115 (34.8%)	20/115 (17.4%)
United States, Wander et al. (2021)	35,879	no diabetes: 22,016	–	–	937/22,016 (4.3%)	1575/22,016 (7.2%)
		diabetes: 13,863	–	–	1 376/13,863 (9.9%)	2 206/13,863 (15.9%)
United States, Barrett et al. (2022)	269,674	no diabetes: 156,982	–	–	66,440/156,982 (42.3%)	19,126/156,982 (12.2%)
		diabetes: 112,692	–	–	59,831/112,692 (53.1%)	20,717/112,692 (18.4%)
France, Bailly et al. (2021)	134,209	no diabetes: 100,200	–	91,298/100,200 (91.1%)	8 902/100,200 (8.9%)	2 568/100,200 (2.6%)
		diabetes: 32,209	–	27,515/32,209 (85.4%)	4 694/32,209 (14.6%)	1 563/32,209 (4.9%)
India, Rangankar et al. (2021)	220	no diabetes: 179	–	153/179 (85.5%)	26/179 (14.5%)	–
		diabetes: 41	–	25/41 (61.0%)	16/41 (39.0%)	–
Japan, Yanagisawa et al. (2021)	131	no diabetes: 107	–	83/107 (77.6%)	24/107 (22.4%)	–
		diabetes: 24	–	4/24 (16.7%)	20/24 (83.3%)	–
Philippines, Espiritu et al. (2021)	10,881	no diabetes: 8 690	mild/moderate: 5 767/8 690 (66.4%)	severe/critical: 2 816/8 690 (32.4%)	1 001/8 690 (11.5%)	1 123/8 690 (12.9%)
		diabetes: 2 191	mild/moderate: 923/2 191 (42.1%)	severe/critical: 1 245/2 191 (56.8%)	739/2 191 (33.7%)	579/2 191 (26.4%)
India, Mithal et al. (2021b)	401	no diabetes: 212	–	severe: 19/212 (8.96%)	26/212 (12.3%)	3/212 (1.4%)
		diabetes: 189	–	severe: 38/189 (20.1%)	46/189 (24.3%)	12/189 (6.3%)
Singapore, Koh et al. (2021)	949	no diabetes: 809	–	severe: 18/809 (2.2%)	5/809 (0.6%)	1/809 (0.1%)
		diabetes: 140	–	severe: 31/140 (22.1%)	21/140 (15.0%)	4/140 (2.9%)

A study from the United States (Chow et al., 2020) described 7,162 cases of SARS-CoV-2 (+) people and showed that diabetes is the most common comorbidity, with a prevalence of 10.9%. The second most prevalent comorbidities were chronic lung disease (9.2%) and cardiovascular disease (9.0%). The hospitalization rate was 4.7-fold higher in diabetic patients compared to healthy people (32 vs 6.8%), while the ICU admission rate was 8.5-fold higher (18.9 vs 2.2%). Further, in the report from France (Bailly et al., 2021), describing 134,209 hospitalized SARS-CoV-2 (+) patients, diabetes was also one of the most common comorbidities (24.0%), after hypertension (49.4%) and just before obesity (23.9%). Both ICU admission and mortality rates were almost 2-fold higher in diabetes patients than in the control population (14.6 vs 8.9% and 4.9 vs 2.6%, for ICU admission and mortality, respectively). In a study from Singapore (Koh et al., 2021), 949 SARS-CoV-2 (+) cases were described, with a severe COVID-19 course occurring 10-fold more frequently in diabetic vs non-diabetic patients (22.1 vs 2.2%). Similarly, the number of diabetic people admitted to ICU was 24-fold higher and the number of deaths in diabetic patients was 23-fold higher, compared to the non-diabetic population (15.0 vs 0.6% for ICU admission and 2.9 vs 0.1% for mortality). In a Chinese study by (Zhang et al., 2020), describing 788 SARS-CoV-2 (+) cases, diabetes was the second most common comorbidity after hypertension, with a prevalence of 5.8%. The number of diabetic patients with a severe course of COVID-19 was 2.6-fold higher compared to the healthy population (14.8 vs 5.7%). Similarly, the critical course of COVID-19 was 39-fold more frequent in people with diabetes, as compared to a healthy population (7.4 vs. 0.2%).

Several studies calculated hazard ratio (HR) of SARS-CoV-2-related death in people with diabetes (Coppelli et al., 2020; Holman et al., 2020; Kim et al., 2020; Al-Salameh et al., 2021; Bowles et al., 2021; Dennis et al., 2021; Diedisheim et al., 2021; Espiritu et al., 2021; Laxminarayan et al., 2021; Mehta et al., 2021; Sonmez et al., 2021; Sutter et al., 2021; Tchang et al., 2021; Woolcott and Castilla-Bancayán, 2021; Yazdanpanah, 2021; Oliveira et al., 2022), demonstrating HR between 1.1 (Diedisheim et al., 2021) and 2.4 (Kim et al., 2021), with an average of 1.6. Further, Espiritu et al. (2021) calculated an odds ratio (OR) of COVID-19-related death in diabetic individuals at 1.5. Surprisingly, in the study of Al-Salameh et al. (2021) COVID-19 mortality in diabetic patients was lower than in general population, with OR of 0.7, yet ICU admission rate was higher, with OR of 2.1.

Together, multiple studies showed that diabetes is one of the most significant comorbidities and is associated with more severe COVID-19 outcome and higher ICU admission and mortality rates in SARS-CoV-2 (+) patients. However, other reports showed that the mortality was comparable in both groups or slightly elevated in people without diabetes. For example, in a French study (Al-Salameh et al., 2021), the mortality was similar for diabetic and non-diabetic patients (21.5 vs 17.4%), yet ICU admission rates still pointed to the worse COVID-19 outcome, with diabetic patients being admitted to ICU 1.5-fold more often (34.8 vs 23.0%). Several other groups reported only a small, 1.2–1.3-fold increase in mortality of diabetic people suffering from COVID-19 (Nikpouraghdam et al., 2020; Borzouei et al., 2021; Ciardullo et al., 2021; Sutter et al., 2021; AbdelGhaffar et al., 2022; Adderley et al., 2022). Moreover, there are reports showing almost no effect or the opposite effect of diabetes on the COVID-19 course (Argenzian et al., 2020; Charlotte et al., 2020; Huang C. et al., 2020; Sutter et al., 2021; Barrett et al., 2022).

There are several reasons for such differences in the severity, hospitalization, and mortality rates. First, the number of people reported within the studies differs significantly, from tens to hundreds of thousands of cases, influencing the statistical results. Second, the demographic status of the populations, such as age, sex, co-existing comorbidities, and socioeconomic status differs between studies, which might impact the results. Other factors would be country-to-country differences in health care quality, availability, criteria for hospital/ICU admission, and prioritization in case of the scarcity of hospital beds. Further, the reporting details make the comparison between the datasets challenging. While the death rates are clearly defined as patients who died with confirmed SARS-CoV-2 infection, the classification of the disease severity is more complicated. Different groups report and categorize data using different systems, from pre-defined official national or international classifications to self-established criteria. Additionally, not all groups included data for patients without any comorbidities which could be considered healthy controls, and comparison to non-diabetic patients might give different results since other comorbidities might exist in those people. Interestingly, in children and adolescents with COVID-19, diabetes is not a comorbidity causing an increased risk for hospitalization due to SARS-CoV-2 infection (d’Annunzio et al., 2020; McGurnaghan et al., 2021; Cardona-Hernandez et al., 2021). Finally, there is a decrease in mortality rate over the time of pandemics, with the earliest reports showing a higher mortality rate difference between diabetic and non-diabetic SARS-CoV-2 (+) patients than the latest studies. The factors accountable for this include higher social awareness, better knowledge about COVID-19 symptoms, diagnosis, and treatment, common vaccinations against SARS-CoV-2, and new variants, which result in the milder course of COVID-19.

Yet, indisputably, the majority of data show that diabetic patients have a worse prognosis when infected with SARS-CoV-2, starting from the disease severity, with more frequent hospitalization and ICU admission, up to increased mortality rates.

## Pancreatitis and New-Onset Diabetes as Complications After SARS-CoV-2 Infection

As the pandemic progresses, an increasing number of reports show that SARS-CoV-2 infection might lead to pancreatic diseases, including acute pancreatitis, ketoacidosis, and new-onset diabetes, in previously healthy individuals with no family history of pancreatic dysfunction.

Pancreatitis is caused by the destabilization of the mechanisms controlling the activity of digestive enzymes, lipase, and amylase, produced by the exocrine pancreas. If enzymes become activated before leaving the pancreas, they directly damage the pancreatic tissue (pancreatic injury) and induce a strong immune reaction (pancreatic inflammation). Untreated, pancreatitis can lead to many complications like fibrosis, cancer, or diabetes, attributing to the loss of pancreatic functions (Petrov, 2021). Mild pancreatitis was reported in 17% of 52 SARS-CoV-2 (+) patients, out of which only 33% were previously diabetic (Wang F. et al., 2020). Similarly, in the study conducted by (Liu et al., 2020), 12 out of 64 COVID-19 patients (19%) were diagnosed with pancreatic injury. SARS-CoV-2-associated pancreatitis was also reported in a Spanish 14-year-old boy (Paz et al., 2021), and, independently, in two out of three members of a Danish family (Hadi et al., 2020). In all cases, other common causes of pancreatic inflammation, such as alcohol or medication consumption, were excluded, indicating idiopathic pancreatitis, which was attributed to the SARS-CoV-2 infection. Pancreatitis occurred infrequently in the study performed by (Inamdar et al., 2020), in which only 189 of 48,012 (0.4%) hospitalized patients met the criteria of pancreatitis diagnosis, yet the infection with SARS-CoV-2 still increased the likelihood more than 3-fold compared to the non-COVID-19 patients.

Ketoacidosis and new-onset diabetes were other reported pancreatic dysfunctions following SARS-CoV-2 infection. Insulin stimulates the glucose uptake from the blood into the cells, and glucose serves as the main source of the energy. In the state of an absolute or relative insulin deficiency the other counterregulatory hormones act to break down the triacylglycerols into free fatty acids, which are further converted into ketones by the liver and stimulate gluconeogenesis. The pathological increase in ketone blood concentration leads to the acidification of the body, called ketoacidosis, which results in serious metabolic complications. Ketoacidosis often accompanies uncontrolled diabetes and requires hospitalization.

In Table 2 we present an overview of the ketoacidosis and new-onset diabetes cases in COVID-19 patients. Generally, the onset of diabetes was acute, with the disease presenting as soon as during SARS-CoV-2 infection (Kuchay et al., 2020; Seow et al., 2021) and up to months after COVID-19 recovery (Xie and Al-Aly, 2022) Patients were diagnosed with diabetes either by routine blood glucose test during COVID-19 infection or on admission to the hospital with ketoacidosis-specific symptoms. Laboratory tests commonly showed increased blood ketone and glucose concentrations, increased glycated hemoglobin A1C (HbA_1C_) level, and a decrease in C-PEPTIDE (C-PEP) or insulin concentration. Of note, in most patients presenting ketoacidosis and new-onset diabetes, COVID-19 symptoms were mild to moderate, with only a few cases with severe infection. Interestingly, in many cases determining the type of diabetes was challenging due to inconclusive symptoms, for example, insulin below detection level was suggestive of T1D, yet islet autoantibodies were not detected. Generally, in most cases, islet autoantibodies were absent, indicating that new-onset diabetes might rather be connected with direct viral damage of pancreatic tissue, rather than activation of autoimmune response.

**TABLE 2:** New-onset diabetes and diabetic ketoacidosis after SARS-CoV-2 infection. T1D–type 1 diabetes; T2D–type 2 diabetes; GAD-65 A–glutamic acid decarboxylase-65 autoantibodies; IA2—anti-islet antigen phosphatase; anti- ZnT8—zinc transporter 8; IAA–antibodies against insulin–no data; M–male; F–female; DKA–diabetic ketoacidosis; NOD–new onset diabetes.

Source	Number of Cases	Age	Sex	HbA_1C_ (%)	Metabolic Complication	Diabetes Type	COVID-19 Clinical Course	C-Peptide (pmol/L)	Islet Auto-Antibodies
ADULTS									
Singapore Chee et al. (2020)	1	37	M	–	ketoacidosis	–	–	–	–
India, Ghosh et al. (2020)	44	mean: 43.3	23 M (52.3%)	mean: 10.2	NOD	–	–	mean: 1900	–
United States , Heaney et al. (2020)	1	54	M	–	ketoacidosis	–	severe (hospitalized)	–	–
Germany, Hollstein et al. (2020)	1	19	M	16.8	ketoacidosis	nonimmune 1B diabetes	mild	210	not detected
China, Li B. et al. (2020)	42 cases out of 658 COVID-19 patients (6.4%); 27 non-diabetic	diabetes mean: 56; no diabetes mean: 41	diabetes: 12 F, 3 M no diabetes: 14 F, 13 M	diabetes: 7.6–12.3 no diabetes: 5.2–5.8	ketosis: 42 patients, ketoacidosis: 5	–	severe (hospitalized)	–	–
India, Kuchay et al. (2020)	3	30	M	9.6	ketoacidosis	–	severe	–	not detected
		60	M	12.6	ketoacidosis	–	moderate	–	not detected
		34	M	12.0	ketoacidosis	–	mild	–	not detected
France, Marchand et al. (2020)	1	29	F	11.8	–	T1D	moderate	0.07	GAD-65 A
India, Ghosh and Misra, (2021)	1	41	M	14.9	hyperglycemia, ketosis	–	mild	480	–
Italy, Montefusco et al. (2021)	65 NOD cases out of 551 COVID-19 patients (11.8%)	–	–	–	hyperglycemia	–	–	–	–
France Potier et al. (2021)	1	31	M	–	ketoacidosis	T1D	severe	–	GAD-65A, ZnT8
India, Sathish and Chandrika Anton, (2021)	21 cases out of 102 COVID-19 patients (20.6%)	median: 50.2	16 M	90% cases: 6.5–8.5	–	–	mild to moderate	–	–
Singapore, Seow et al. (2021)	3	9	M	>15	ketoacidosis	nonimmune 1B diabetes	mild	207	not detected
		30	M	11.8	ketoacidosis	nonimmune 1B diabetes	mild	282	not detected
		48	M	11.1	ketoacidosis	nonimmune 1B diabetes	mild	349	not detected
United States , Suwanwongse and Shabarek, 2021)	2	51	M	12.4	ketoacidosis, ketonemia, hyperglycemia	–	mild	–	–
		64	F	–	ketonuria, hyperglycemia	–	mild	–	–
Italy, Tollard et al. (2021)	1	32	F	13.1	ketoacidosis	–	severe	–	IA2
Australia, Venkatesh et al. (2021)	1	45	M	16.2	ketoacidosis	T1D	severe (hospitalized)	–	not detected
Egypt, Motawea et al. (2022)	1	73	F	–	polyuria, polydipsia	T2D	–	–	–
United States , Patel et al. (2021)	1	44	F	–	DKA	–	severe	–	–
United States , Alshamam et al. (2021)	1	20	M	13.7	polyuria, polydipsia	–	mild	200	–
CHILDREN									
United Kingdom, Unsworth et al. (2020)	5	median: 11.6	2 F 3 M	median: 3.3	ketoacidosis, DKA	T1D	–	–	–
Egypt, Alfishawy et al. (2021)	1	17	M	14.7	DKA	T1D	severe	–	GAD-65 A, IA2
Morocco, Aabdi et al. (2021)	1	3	M	10	ketoacidosis	–	–	–	–
Marocco, Benyakhlef et al. (2021)	1	3	M	10.3	DKA	–	severe	–	GAD-65 A
United States , Naguib et al. (2021)	1	8	F	12	hyperglycemia, DKA	–	severe	–	not detected
United States , Suwanwongse and Shabarek, 2021)	1	18	M	10.4	ketoacidosis	–	asymptomatic	–	not detected
United States , Alizadeh et al. (2021)	1	16 months	M	9.5	DKA	T1D	moderate	–	ZnT8, GAD-65 A, IA2
United States , Basta et al. (2021)	1	3	M	–	hyperlipidemia, DKA	T1D	mild	–	GAD-65 A, IAA
India, Parappil et al. (2022)	1	12	M	–	DKA	–	critical	–	–
Portugal Lança et al. (2022)	2	13	M	15.3	polyphagia, polyuria, ketonemia, hyperglycemia, DKA	–	mild	130	GAD-65 A
	8	M	12.4	ketonemia, hyperglycemia, DKA	–	mild	undetected	GAD-65 A
United States , Barrett et al. (2022)	IQVIA 68 NOD cases of 80,893 COVID-19 patients (0.1%)	mean: 12.3	34 F (50%)	–	NOD, DKA	–	–	–	–
	Healtherity 1 120 NOD cases of 280,767 COVID-19 patients (0.4%)	mean: 12.7	602 F (54%)	–	NOD, DKA	–	–	–	–
United States , Ambati et al. (2022)	2	10	F	11.9	polydipsia, polyuria, DKA	–	asymptomatic	–	not detected
		17	F	14.8	polydipsia, polyuria, DKA	–	asymptomatic	–	–
Turkey, Ata et al. (2022)	57 out of 118 COVID-19 patients (48%)	6–18	–	11.4	63.2% DKA	T1D	asymptomatic	median: 90	GAD-65 A–37 (65%), IAA—25 (43%); IAA and GAD-65 A–15 (26%)

Out of the publications presented in Table 2 only several studies (Ghosh et al., 2020; Li J. et al., 2020; Montefusco et al., 2021; Sathish and Chandrika Anton, 2021; Barrett et al., 2022) reported a larger sample size, and the rest are case reports. Hollstein et al. (2020) reported a case of a 19-year-old, previously healthy man with a body mass index (BMI) of 22.6 kg/m^2^ who was diagnosed with new-onset autoantibody-negative, insulin-dependent diabetes. Diabetes was previously present in his family. A similar case was observed with a man with no previous familial diabetes and a normal BMI (21.3 kg/m^2^). Three weeks after the SARS-CoV-2 infection, he developed hyperglycemia and ketoacidosis, and was diagnosed with T1D (Ghosh et al., 2020).

Many new-onset diabetes cases were reported also in children. Basta et al. (2021) reported a case of 3-year-old male admitted with hyperlipidemia, hyperglycemia, and diabetic ketoacidosis (DKA) and diagnosed with T1D within 11 days after infection with SARS-CoV-2. Further, a 17-year-old male with COVID-19 was hospitalized with DKA and pancreatitis symptoms, and diagnosed with T1D (Alfishawy et al., 2021). Unsworth et al. (2020) assessed five SARS-CoV-2 (+) children with a median age of 11.6. Four out of five children (80%) presented with DKA, including three with severe DKA. Barrett et al. (2022) showed that new-onset diabetes was 166% (source: IQVIA^#^) and 31% (source: HealthVerity^##^) more frequent among children and adolescent patients with COVID-19 than among those without COVID-19 compared to the healthy population. In a study conducted in India (Sathish and Chandrika Anton, 2021) 21 out of 102 patients (20.6%) were diagnosed with new-onset diabetes while admitted with mild to moderate COVID-19. This study shows a surprisingly high rate of new-onset diabetes accompanying SARS-CoV-2 infection. Among these 21 patients, all had elevated fasting plasma glucose and 90% had elevated HbA_1C_ levels. Since HbA_1C_ is an indicator of long-term glycemic control, it suggests that a high number of new-onset diabetes in this study might, at least partially, reflect the previously undiagnosed diabetes, existing before SARS-CoV-2 infection.

SARS-CoV-2 may induce diabetes directly by infecting β-cells or indirectly, for instance, inflammation can impair glucose metabolism, causing insulin resistance or increased hepatic glucose production (Accili, 2021). Independently of mechanism, diabetes or impaired glucose metabolism might occur within the weeks or months post SARS-CoV-2 infection. Thus, the full effect of SARS-CoV-2 infection will be observed in the long term. The effect of viral infection on the destruction of β-cells has been previously demonstrated in SARS-CoV(+) patients. Interestingly, in the follow-up study conducted 3 years later, insulin levels in the group that developed diabetes after SARS-CoV infection were similar to the healthy population, which suggests that the pancreatic cell damage by SARS-CoV was temporal (Yang et al., 2010). As for SARS-CoV-2, so far there are only a few follow-up reports, showing conflicting results. For instance, Seow et al. (2021) reported three cases of patients diagnosed with non-immune T1D after COVID-19. On admission, patients developed ketoacidosis and were started on insulin therapy. Two months later, the level of C-PEP in all three cases was still pathologically lowered, and patients were still insulin-dependent. Conversely, in the follow-up performed by Kuchay et al. (2020) after 14 weeks from SARS-CoV-2-associated diabetes onset, insulin levels were stabilized and patients were taken off the insulin, while only maintaining metformin therapy. Montefusco et al. (2021) showed that among the group of 57 patients with new-onset hyperglycemia detected at admission with SARS-CoV-2 infection, 35% were still hyperglycemic after 6 months, 2% were diagnosed with diabetes, and in the remaining patients, blood glucose level returned to normal. Similarly, out of 64 SARS-CoV-2 (+) patients with new-onset diabetes, 56% were hyperglycemic after 323 days, while 41% returned to a normoglycemic state (Cromer et al., 2022). Gupta et al. examined 19 SARS-CoV-2 (+) patients with new-onset islet autoantibody (-) diabetes. After 6 months, 79% of the patients achieved proper glycemic control and were taken off insulin therapy, while 21% remained insulin-dependent. Four months later (10 months total), 79% of the patients were still normoglycemic despite the lack of insulin treatment (Gupta et al., 2022). Long-term follow-up, with a larger sample size, could clarify the degree and timeframe of the reversibility of SARS-CoV-2-mediated pancreatic damage.

Additionally, it is worth noticing that multiple factors can contribute to the development and severity of both diabetes and COVID-19. For instance, disturbances in gut microbiome composition and gut permeability might underlie a low-grade inflammation, which has been linked to new-onset diabetes (Alkanani et al., 2015; Leiva-Gea et al., 2018; Zhang et al., 2022) and to the increased severity and mortality of COVID-19 (Giron et al., 2021; Giannos and Prokopidis, 2022; Schult et al., 2022). Similarly, diabetes and SARS-CoV-2 infection both trigger pro-inflammatory reactions independently. State of inflammation induces changes in tryptophan conversion, increasing production of aryl hydrocarbon receptor (AhR) activators, including kynurenine (Salminen, 2022) and indole-3-pyruvate (I3P). AhR activation dysregulates immune response, contributing to the cytokine storm, a response closely connected with COVID-19 severity. Additionally, changes in tryptophan metabolism reduce the production of melatonin, a potent antioxidant agent, further enhancing the state of inflammation. For more details, visit a review of Anderson et al. (Anderson and Reiter, 2020; Anderson et al., 2021). Hasan et al. (2022) showed that melatonin treatment reduces thrombosis, sepsis, and mortality rates in COVID-19 patients. AhR, tryptophan metabolism pathway, and melatonin levels are also implicated in β-cell and pancreatic function (Yue et al., 2020). Dioxin-induced AhR activation is associated with mitochondrial dysfunction, oxidative stress, insulin secretion inhibition, and pancreatic cell death in rodents (Martino et al., 2013; Ghosh et al., 2018). Further, treatment with melatonin in rat INS-1 cell line has a protective effect against hyperglycemia-induced β-cell stress and improves insulin synthesis and insulin secretion (Jung et al., 2020; Lee et al., 2020). Overall, these data suggest that there might be a connection between diabetes, SARS-CoV-2 infection, gut dysbiosis, and AhR receptor, yet more studies in human settings are needed to establish the direct links.

^#^IQVIA–database of health care reports from closed U.S. health plans, including reports from primarily commercial health plans used to provide a complete view of patient care across all care settings. The study period was from 29 January 2019, to 31 March 2021, for the pandemic period groups and 29 January 2016–31 March 2018, for the pre-pandemic period groups.

^##^HealthVerity–database that provides access to patient-level linked data from 70 different commercial health data sources using privacy-preserving record linkage to generate a comprehensive and longitudinal patient history. The study period was 1 December 2018–31 July 2021.

## SARS-CoV-2 Entry Into Host Cells

The Spike (S)-protein of the viral capsid plays the main role in the process of the infection by SARS-CoV-2, just as in SARS-CoV and MERS-CoV. To penetrate the host cell, coronavirus S-protein must bind to its receptor on the cell surface. The high sequence similarity of S-protein between SARS-CoV-2, SARS-CoV, and MERS-CoV (Xu et al., 2020) suggests that all three viruses could use the same receptors for cell entry. The main receptor for SARS-CoV-2 entry into the cells is ACE2, just as in SARS-CoV, however, MERS-CoV uses primarily DPP4 receptor. Following the binding of SARS-CoV-2 S-protein with ACE2 receptor, FURIN cleaves the boundary between S1 and S2 subunits of S-protein, which enables the conformational change and exposure of the cleavage site within the S2 subunit. Cleavage of this site by proteases is necessary for the next step - virus fusion with the cell. Depending on the protease type present in the host cell, there are different modes of virus entry. If no protease is present on the cell surface, the ACE2-virus complex is internalized *via* endocytosis. Inside the endolysosome, the S2 subunit is cleaved by cathepsin L (CTSL), which leads to the fusion of endolysosome and viral membranes, and viral RNA release. Alternatively, transmembrane serine protease 2 (TMPRSS2), a cell membrane-bound protease, might facilitate the S2 subunit cleavage. This allows the fusion of cell and viral membranes without endocytosis, and a release of the viral RNA into the cell (Geravandi et al., 2021; Jackson et al., 2022).

Growing evidence suggests that there might be other receptors involved in SARS-CoV-2 entry into the host cell. First, ACE2 expression alone does not explain a wide range of infected tissues and cell types, some of which exhibit very low or no expression of this receptor (Chua et al., 2020; Hikmet et al., 2020; Ren et al., 2021). Second, despite ACE2 being a common receptor for both SARS-CoV-2 and SARS-CoV, there are significant differences in cell tropism, replication, and cell damage profile between those viruses (Chu et al., 2020). Third, the treatment with ACE2-blocking antibodies significantly reduces but does not completely abrogate SARS-CoV-2 entry into the host cell (Chen et al., 2021).

One of the SARS-CoV-2 alternative receptors is the transmembrane glycoprotein CD147, which was previously found to mediate SARS-CoV infection (Chen et al., 2005). CD147 directly interacts with SARS-CoV-2 S-protein *in vitro* as shown by surface plasmon resonance and enzyme-linked immunosorbent assay (ELISA). Moreover, these two proteins colocalize in the Vero E6 monkey kidney cell line, a cell line commonly used for SARS-CoV studies, as well as in kidney and lung tissue from COVID-19 patients, suggesting their interaction also *in vivo*. CD147 stable knockdown in monkey kidney and human lung cell lines significantly decreases SARS-CoV-2 copy number, while overexpression promotes virus entry in these cells. Additionally, CD147 efficiently facilitates SARS-CoV-2 infection in the ACE2-deficient cells, pointing at the ACE2-independent SARS-CoV-2 entry. Upon binding to the CD147 receptor in Vero E6 cells, SARS-CoV-2 is internalized via endocytosis (Wang K. et al., 2020; Masre et al., 2021; Jackson et al., 2022).

Another putative receptor involved in SARS-CoV-2 entry into the cell is Neuropilin 1 (NRP1). NRP1 directly binds the S1 subunit of S-protein, which is cleaved by FURIN upon SARS-CoV-2 binding to the ACE2 receptor (Daly et al., 2020). This binding accelerates the separation of S1 and S2 subunits, and exposition of S2 cleavage site to TMPRSS2, facilitating SARS-CoV-2 internalization (Li and Buck, 2021). While NRP1 alone is not sufficient for SARS-CoV-2 infection of HEK293T cells, it potentiates infectivity when co-expressed with ACE2 and TMPRSS2, suggesting that NRP1 might act as a cofactor and enhancer of ACE2-dependent entry mechanism (Cantuti-Castelvetri et al., 2020).

The DPP4 was previously shown to allow the infection of the human respiratory tract by MERS-CoV (Meyerholz et al., 2016). DPP4 and ACE2 expression patterns show similarity, suggesting DPP4 involvement in ACE2-dependent SARS-CoV-2 infection (Qi et al., 2020; Venkatakrishnan et al., 2020). Additionally, *in silico* crystallographic analysis demonstrated the SARS-CoV-2 affinity to the DPP4 receptor (Li Y. et al., 2020). Yet, there are some contradictory results, suggesting that DPP4 and SARS-CoV-2 do not interact *in vitro* (Xi et al., 2020). Therefore, the DPP4 role in SARS-CoV-2 entry to the cell requires clarification.

Recently, genomic receptor profiling allowed the identification of asialoglycoprotein receptor-1 (ASGR1) and kringle containing transmembrane protein (KREMEN1) as membrane proteins interacting with SARS-CoV-2 S-protein, suggesting they might be alternative SARS-CoV-2 receptors. Both ASGR1 and KREMEN1 mediate SARS-CoV-2 infection in ACE2 knockout HEK293T cells. Also, in mouse models, the expression of human ASGR1 and KREMEN1 allows SARS-CoV-2 entry. However, both in *vitro* and *in vivo* settings*,* the infection via ASGR1 or KREMEN1 is not as efficient as the one mediated by ACE2. Interestingly, ASGR1 and KREMEN1 are specifically promoting SARS-CoV-2 but not SARS-CoV entry. Additionally, a characteristic combination of all three receptors seems to be used by SARS-CoV-2 to infect different cell types, potentially being responsible for its multi-organ infection pattern (Masre et al., 2021; Gu et al., 2022; Hoffmann and Pöhlmann, 2022).

The mode of SARS-CoV-2 entry into the cells seems to be strongly tissue-specific, while ACE2 remains the main receptor for the virus, other proteins might enhance ACE2 function or even function as independent receptors in the case of ACE2 deficiency. Research on the possible receptors and co-receptors for SARS-CoV-2 is still ongoing and we might expect additional molecules to be involved in SARS-CoV-2 entry into the host cell.

## Approaches to Studying SARS-CoV-2 Infection in the Human Context

The major approaches used to study SARS-CoV-2 infection in human cells or organs include examination of the fixed tissues from COVID-19 patients, culturing patient primary cell-derived or human pluripotent stem cell-derived 3D organoids and spheroids, and *in vivo* models, like the humanized mice. Several crucial elements should be considered while investigating SARS-CoV-2 infection: 1) cell susceptibility to the infection, 2) virus entry and replication, and 3) the morphological and functional consequences of the infection. Organ or cell type susceptibility to the infection might be assessed by the evaluation of the expression of molecular factors that permit virus entry into the host cell, including receptors and auxiliary proteins. The presence of the proteins involved in SARS-CoV-2 entry is commonly assessed by the post-mortem examination of fixed tissue obtained from deceased COVID-19 patients or the examination of cell cultures, 3D organoids, or hPSC-derived spheroids *in vitro*. The *in vitro* cell culture condition might impact the expression of SARS-CoV-2-associated proteins. However, the receptor presence is not synonymous with effective infection, and conversely, the lack of known receptors does not imply that the cell cannot be infected. Fixed tissues allow the *ex vivo* evaluation of the infection in a particular cell type by staining against the viral double-stranded RNA (dsRNA) or the components of the viral capsid, for example, S-protein or nucleocapsid (N)-protein. Still, these methods suffer major limitations, most importantly, the lack of insight into the dynamics of the virus entry, replication, and the influence on cell biology. Moreover, additional comorbidities or pre-existing conditions in patients might overlap with the effects of COVID-19, making it challenging to recognize the changes specific to the SARS-CoV-2 infection.

The dynamics of SARS-CoV-2 infection can be investigated in the *in vitro* systems. Immortalized cell lines are long-lived, easily obtained, and provide a wide variety of cell types, however, since they are either cancer cells lines or cells immortalized artificially, they might behave differently than primary cells. Also, they are usually homogenous cultures that lack the interactions between closely located cell types, which are characteristic of the native tissue. Human primary cells obtained from patients present some cell heterogeneity, yet, they are difficult to access and often can only be cultured for a limited number of passages, except for adult stem cells or progenitors. The hPSC differentiation provides an almost unlimited source of different human cell types to study the SARS-CoV-2 infection and its effect on cell survival, differentiation, and function. During differentiation, hPSCs are guided through the consecutive stages, recapitulating the developmental processes. Consequently, they can differentiate into multiple cell types that often closely resemble native cells in terms of gene expression, morphology, and functionality.

Both primary cells and hPSC-derived cells can be cultured in 3D format, as organoids and spheroids, respectively. 3D cultures recapitulate the native tissue better than two-dimensional (2D), due to their complex cytoarchitecture and the heterogeneity of cell types found within a particular organ. This provides a specific microenvironment in which different cell types, influence and regulate each other’s functions. 3D *in vitro* systems are widely used for a broad range of applications, including gene expression analysis to seek potential SARS-CoV-2 receptors, SARS-CoV-2 infection dynamics, and its functional consequences, as well as for drug testing. 3D cell clusters were successfully used for studies on SARS-CoV-2 infection in the lung (Huang J. et al., 2020; Youk et al., 2020), heart (Yang et al., 2020; Marchiano et al., 2021) intestine (Lamers et al., 2020; Mithal et al., 2021a), brain, kidney (Jansen et al., 2022), liver (Yang et al., 2020; Zhao et al., 2020) and pancreas (Yang et al., 2020). The *in vitro* obtained data on virus entry or SARS-CoV-2 impact on cell biology, need to be confirmed *in vivo*.

Compared to the *in vivo* animal models, the 3D *in vitro* systems do not give a complete picture of the infection, for example, they lack complete immune system response, such as infiltration of the infected tissue by immune cells. While primates are susceptible to the SARS-CoV-2 infection, their use as model animals is limited. Since mice are not susceptible to the infection with SARS-CoV-2, humanized mice which carry functional human genes, cells, or tissues, are used as a cost-effective, accessible alternative. In this model, only human tissues or cells but not mouse cells would be infected. Humanized mice have been for example used as a platform for research on SARS-CoV-2 receptor-neutralizing antibodies and other drug discovery and testing (Hansen et al., 2020; Du et al., 2021), however, they could also be used to study the infection course and complications (Sefik et al., 2021; Wu M.-L. et al., 2021).

## Expression of SARS-CoV-2 Receptors in the Pancreas

**FIGURE 1:**
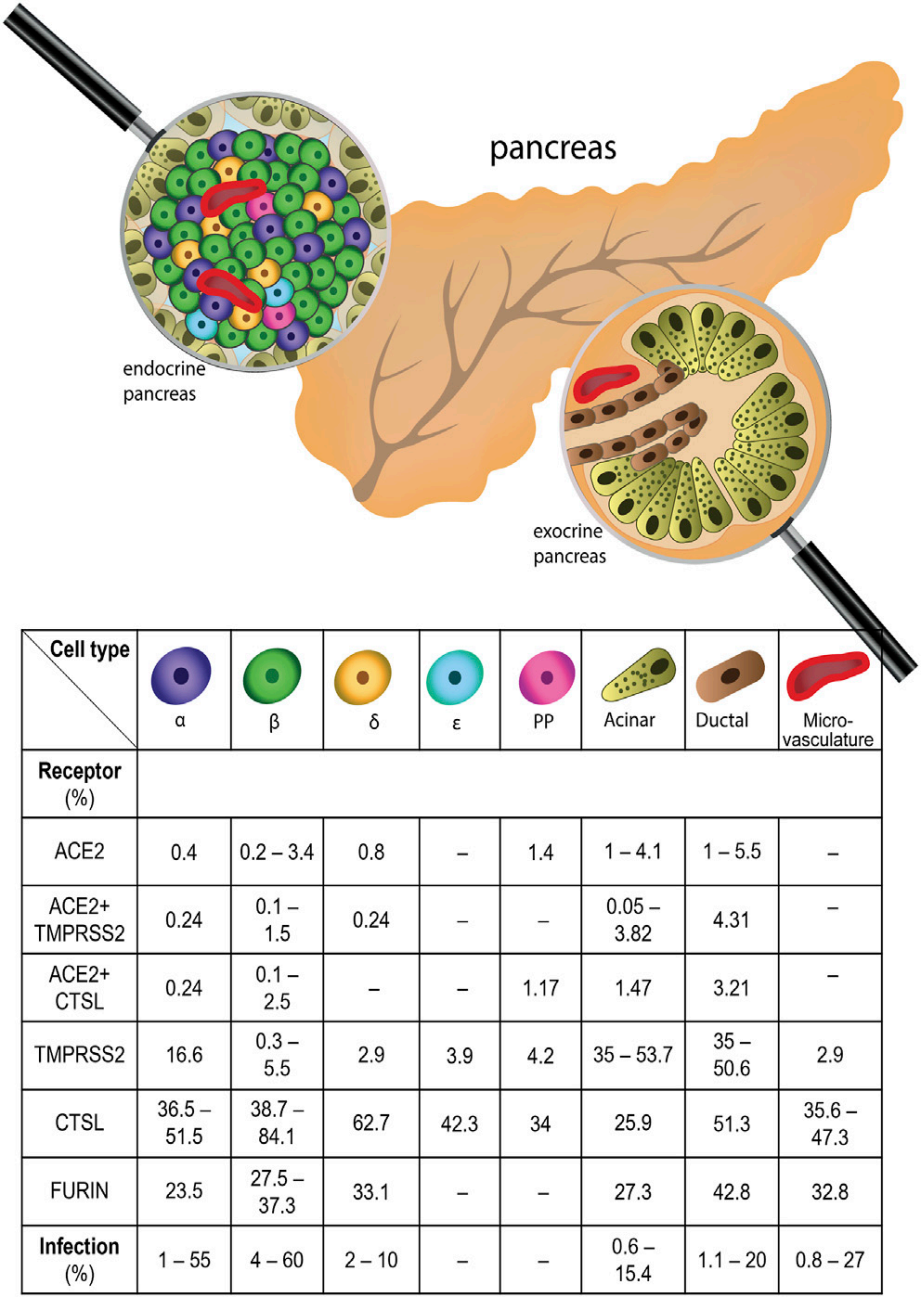
Expression of SARS-CoV-2 receptors in human pancreas and frequency of SARS-CoV-2 infection in pancreatic cells. All major pancreatic cell types express SARS-CoV-2 receptors, with varying frequency. The percentages of cells expressing SARS-CoV-2 receptor mRNAs, including ACE2 - Angiotensin converting enzyme 2, NRP1 - Neuropilin 1, TMPRSS2 - Transmembrane Serine Protease 2, CTSL - Cathepsin L in different types of pancreatic cells is shown. Additionally, the percentage of the cells infected by SARS-CoV-2 in certain pancreatic cell types is shown.

Clinical data show that pancreatitis, ketoacidosis, and new-onset diabetes might accompany COVID-19, suggesting that pancreatic cells might be infected by SARS-CoV-2. To delineate the relationship between COVID-19 and diabetes, it is crucial to assess whether pancreatic cells, especially β-cells, can be directly infected by SARS-CoV-2. The presence of the molecules involved in the SARS-CoV-2 entry, including ACE2, TMPRSS2, FURIN, CTSL, NRP1, and DPP4 has been studied in human pancreatic cells (Figure 1). In this chapter, we review in detail the presence of SARS-CoV-2 receptors in the pancreatic cell types.

### β-cells

ACE2 and TMPRSS2 mRNAs were detected by RT-qPCR in EndoC-βH1, a model human β-cell line (Fignani et al., 2020; Müller et al., 2021), with ACE2 expression level similar to human primary islets (Fignani et al., 2020). The scRNA-seq data show low expression of ACE2 and TMPRSS2 in β-cells. Analysis of three publicly available scRNA-seq datasets revealed low levels of ACE2 and TMPRSS2 (Wu C.-T. et al., 2021) and examination of another four scRNA-seqs showed that less than 1.5% of β-cells express either mRNA (Coate et al., 2020). ScRNA-seq performed by Yang et al. (2020) identified ACE2 and TMPRSS2 expression in a small portion of β-cells. Kusmartseva et al. (2020) analyzed scRNA-seq data from 22 healthy patients to find that only 0.3% of β-cells coexpress ACE2 and CTSL and 0.1% coexpress ACE2 and TMPRSS2. Coate et al. (2020) did not find any β-cells coexpressing ACE2 and TMPRSS2. Visualization of mRNA expression pattern *in situ* in fixed samples from seven healthy individuals showed ACE2 and TMPRSS2 mRNAs in only a few endocrine cells, with almost no colocalization with INS (Kusmartseva et al., 2020). At the protein level, ACE2 and SARS-CoV-2 cofactor proteins were detected in β-cells, with varying frequency and level. ACE2 and TMPRSS2 proteins were detected by immunoblotting and immunostaining in the EndoC-βH1 cell line (Fignani et al., 2020; Müller et al., 2021). Of note, Fignani et al. (2020) showed a differential distribution of ACE2 isoforms, suggesting that the short isoform is prevalent in EndoC-βH1 cells. ACE2 and TMPRSS2 protein presence were variable in immunostainings of pancreatic fixed tissues, with some publications showing various levels of their expression in β-cells (Fignani et al., 2020; Yang et al., 2020; Müller et al., 2021; Qadir et al., 2021; Steenblock et al., 2021; Wu C.-T. et al., 2021), while others reported no detectable ACE2 protein in INS(+) cells (Coate et al., 2020; Kusmartseva, et al., 2020). Examination of tissues from five healthy subjects showed that ACE2 and TMPRSS2 proteins are both expressed in islet cells, with ACE2 being expressed 2-fold, and TMPRSS2 3-fold more frequently in β-cells than in α- and δ-cells (Müller et al., 2021). Similarly, Wu C.-T. et al. (2021) demonstrated the low levels of both ACE2 and TMPRSS2 in β-cells in five healthy individuals. Further, Fignani et al. (2020) reported a weak, diffused expression of ACE2, which highly colocalized with INS in seven healthy donors. Steenblock et al. (2021) found a high expression of ACE2 protein in β-cells but only in one out of three SARS-CoV-2 (-) samples. Fadh Qadir et al. (Qadir et al., 2021) reported that ACE2 protein was expressed in ∼6% of INS(+) cells in tissue sections from eight subjects. In human primary islets, ACE2 protein expression in β-cells was also confirmed (Yang et al., 2020). Contrarily, no ACE2 (+)/INS(+) cells were found in the studies by Coate et al. (2020), Kusmartseva, et al. (2020) in neither adult and juvenile pancreas sections nor in human primary islets. Also, TMPRSS2 was not detected in β-cells in the study by Coate et al. (2020). Interestingly, Coate et al. (2020) used the same ACE2 antibody as Yang et al. (2020) and three additional antibodies, yet the groups obtained conflicting results. Yang et al. (2020) found the ACE2 protein in hPSC-derived β-cells. β-cells were transplanted into immunocompromised SCID-beige mice and ACE2 expression was also found in human β-cells 2 months post-transplantation (Yang et al., 2020).

Expression of FURIN mRNA was found in β-cells in the study of Wu C.-T. et al. (2021) which included data from three independent scRNA-seqs. FURIN protein expression in β-cells was examined yet not identified in the healthy tissues of pancreatic adenocarcinoma surgical margin from six individuals (Portela-Gomes et al., 2008).

Another SARS-CoV-2 receptor, NRP1, was detected in β-cells at mRNA and protein levels (Wu C.-T. et al., 2021). Analysis of three scRNA-seq datasets showed an abundant NRP1 mRNA expression in β-cells (Wu C.-T. et al., 2021). At the protein level, NPR1 was detected in a high percentage of β-cells, in contrast to a small portion of NRP1 (+) α-cells in five healthy donors (Wu C.-T. et al., 2021). Hasan et al. (2010) also found NRP1 protein expression colocalizing with INS. Further, Tang et al. (2021) demonstrated a high degree of NRP1 and INS colocalization in autopsy tissues using two different antibodies.

DPP4 expression was identified in β-cells. At the mRNA level, the RNA-seq of EndoC-βH1 cells showed high DPP4 expression (Bugliani et al., 2018). As for the protein, Bugliani et al. (2018) reported that 29% of INS(+) cells also expressed DPP4 in the tissues of four healthy patients. In contrast to that, a minimal INS and DPP4 colocalization (Omar et al., 2014) or no colocalization (Augstein et al., 2015; Coate et al., 2020) was reported in immunostained tissues from non-diabetic patients.

### α-cells

ACE2 and SARS-CoV-2 proteases, including TMPRSS2, CTSL, and FURIN are expressed at low levels in human α-cells. As by the analysis of scRNA-seq data from 22 nondiabetic donors, only 0.2% of α-cells had ACE2 and TMPRSS2 and 0.2% had ACE2 and CTSL expression (Kusmartseva, et al., 2020). Similarly, the analysis of the three scRNA-seq datasets performed by Wu C.-T. et al. (2021) confirmed the low level of ACE2 and TMPRSS2 mRNAs in α-cells but showed abundant FURIN expression in α-cells. Further, scRNA-seq of dispersed human primary islets revealed rare ACE2 (+) and some TMPRSS2(+) α-cells (Yang et al., 2020). Four other scRNA-seq datasets showed almost no expression of ACE2 and TMPRSS2 in α-cells, yet low but widespread CTSL expression was found (Coate et al., 2020). There are also conflicting results concerning ACE2 and TMPRSS2 protein expression in α-cells. Coate et al. (2020) found no detectable ACE2 protein in neither post-mortem tissue sections, nor in primary human islets using four different antibodies. Fignani et al. (2020) found weak ACE2 expression rarely colocalizing with GCG in fixed tissues from seven healthy subjects. Further, Müller et al. (2021) detected both ACE2 and TMPRSS2 proteins in α-cells, with a frequency comparable to δ-cells but lower than in β-cells in five healthy donors. Immunostaining of the pancreas from eight donors showed the ACE2 protein in ∼17% of GCG (+) cells (Qadir et al., 2021). Wu C.-T. et al. (2021) found ACE2 and TMPRSS2 expression in a large portion of α-cells in fixed pancreas from five non-COVID-19 patients. Further, Yang et al. (2020) detected a widespread ACE2 protein expression within α-cells of human primary islets and in hPSC-derived α-cells (Yang et al., 2020). Additionally, 2 months after the transplantation of hPSC-derived endocrine cells into mice, ACE2 protein was still present in GCG (+) cells (Yang et al., 2020).

FURIN mRNA expression was found in α-cells in the analysis of the three independent scRNA-seq datasets (Wu C.-T. et al., 2021). Expression of FURIN protein was detected in a subset of GCG (+) cells in immunostaining of the healthy pancreatic tissues from six patients (Portela-Gomes et al., 2008).

NRP1, another receptor for SARS-CoV-2 entry, was abundantly expressed in α-cells at the mRNA level in the analysis of the three scRNA-seq datasets (Wu C.-T. et al., 2021). Immunostaining in samples from five healthy patients detected NRP1 protein in a small percentage of α-cells (Wu C.-T. et al., 2021). Further, NRP1 (+)/GCG (+) cells were also detected in the immunostaining performed by Hasan et al. (2010).

DPP4 protein expression in α-cells was reported by several sources. Omar et al. (2014) showed that in sections from healthy individuals DPP4 protein is present almost exclusively in α-cells. DPP4 expression was identified in 91% of GCG (+) cells, as assayed by the immunostaining of the tissues from four healthy patients (Bugliani et al., 2018).

### δ-cells

Only a few publications showed ACE2 expression in δ-cells. An analysis by Kusmartseva et al. (2020) of scRNA-seq datasets from 22 healthy subjects showed that only 0.2% δ-cells are ACE2 (+)/TMPRSS2(+) and 0.2% are ACE2 (+)/CTSL (+). On the protein level, ACE2 and TMPRSS2 were detected in SST (+) δ-cells, with a lower frequency than in INS(+) β-cells and comparable frequency to GCG (+) α-cells (Müller et al., 2021). However, in hPSC-derived δ-cells, no ACE2 (+) cells were identified (Yang et al., 2020).

FURIN mRNA expression in δ-cells was detected in one out of three scRNA-seq datasets in the analysis by Wu C.-T. et al. (2021). Protein expression of FURIN was examined by Portela-Gomes et al. (2008) yet no FURIN(+)/SST (+) cells were found.

Protein expression of DPP4 in δ-cells was assessed by several studies, however, none of them detected DPP4 in SST (+) cells (Omar et al., 2014; Bugliani et al., 2018).

### Acinar Cells

*In situ* hybridization of mRNA in pancreata from seven healthy donors confirmed the expression of ACE2 and TMPRSS2 in acinar cells (Kusmartseva, et al., 2020). Some publications showed that ACE2 expression in acinar cells is rather rare, while the TMPRSS2 expression is more widespread (Coate et al., 2020; Yang et al., 2020). Pooled analysis of four scRNA-seq data showed that only 1% of exocrine cells express ACE2, while as much as 35% express TMPRSS2 (Coate et al., 2020). ScRNA-seq performed by Yang et al. (2020), showed a similar pattern of infrequent ACE2 and broader TMPRSS2 expression within acinar cells. Shaharuddin et al. (2021) found that ACE2 and TMPRSS2 were expressed in hPSC-derived acinar cells, but their levels were significantly lower than in human primary acinar cells. The integrated analysis of scRNA-seq data from 22 non-diabetic donors revealed that 3.8% of acinar cells coexpress ACE2 and TMPRSS2, while 1.5% coexpress ACE2 and CTSL, suggesting that TMPRSS2-mediated, endocytosis-independent mode of virus entry is dominant in acinar cells (Kusmartseva, et al., 2020). Interestingly, in diabetic donors, the percentage of the cells coexpressing ACE2 with TMPRSS2, and ACE2 with CTSL, was increased 2-fold and almost 5-fold, respectively (Kusmartseva, et al., 2020).

As for protein, Müller et al. (2021) showed a weak ACE2 and barely detectable TMPRSS2 staining in GATA4 (+) acinar cells of five healthy subjects. Also, Shaharuddin et al. (2021) observed ACE2 and TMPRSS2 protein expression in hPSC-derived acinar cells.

### Duct Cells

Similarly to acinar cells, in ducts, ACE2 expression is also less frequent than TMPRSS2. A cumulative analysis of scRNA-seq data from four independent datasets showed that less than 1% of exocrine cells express ACE2, while as much as 35% express TMPRSS2 (Coate et al., 2020). Concomitantly, scRNA-seq of human primary tissue revealed ACE2 expression in low and TMPRSS2 expression in a higher percentage of ductal cells (Yang et al., 2020). Additionally, *in situ* hybridization confirmed ACE2 and TMPRSS2 expression in ducts, with TMPRSS2 being more frequent (Kusmartseva, et al., 2020). Surprisingly, in hPSC-derived ducts, an inverse observation was made - a high level of ACE2 mRNA and a low level of TMPRSS2 mRNA was detected (Shaharuddin et al., 2021). An integrative analysis of scRNA-seq data from 22 non-diabetic donors demonstrated 4.3 and 3.2% of ACE2 (+)/TMPRSS2(+) and ACE2 (+)/CTSL (+) ducts, respectively (Kusmartseva, et al., 2020). Many studies confirmed the ACE2 protein expression in ducts based on the immunostaining of the pancreas from healthy patients. ACE2 was widely and consistently detected in ducts using four different antibodies (Kusmartseva, et al., 2020). Immunostaining of the fixed tissues from eight donors showed that ∼22% of KRT19(+) cells coexpress ACE2 (Qadir et al., 2021). In contrast, Fignani et al. (2020) identified only rare scattered ACE2 (+) ducts. Further, Müller et al. (2021) detected ACE2 in KRT19(+) cells, with varying expression levels. TMPRSS2 was also observed in some ductal cells (Müller et al., 2021). Coate et al. (2020) showed that while ACE2 and TMPRSS2 proteins are present in KRT19(+) ducts, they are rarely coexpressed. Both ACE2 and TMPRSS2 proteins were also present in KRT19(+) hPSC-derived ducts (Shaharuddin et al., 2021).

DPP4 protein was detected in 31% of CD133 (+) ducts, as assayed by the flow cytometry of dispersed human pancreatic tissues (Augstein et al., 2015).

### Pancreatic Microvasculature

Multiple studies showed the ACE2 presence in pancreatic microvasculature at both mRNA and protein levels. In the scRNA-seq analysis of primary human islets (Yang et al., 2020) and 22 whole-pancreas healthy donors (Kusmartseva, et al., 2020), ACE2 was not detected in endothelial cells, while TMPRSS2 and CTSL were found in 2.9 and 35.3% of endothelial cells. Further, *in situ* mRNA hybridization performed on autopsy tissues from seven non-diabetic, SARS-CoV-2 (-) patients, showed a widespread TMPRSS2 and rare ACE2 expression within CD34 (+) endothelial cells (Kusmartseva, et al., 2020). At the protein level, several reports showed ACE2 expression in pancreatic microvascular structures by immunostaining of tissue sections from healthy donors using various anti-ACE2 antibodies (Coate et al., 2020; Fignani et al., 2020; Kusmartseva, et al., 2020; Müller et al., 2021). ACE2 signal was associated but not colocalized with endothelial cell marker, CD31 (Coate et al., 2020; Fignani et al., 2020). Detailed analysis showed ACE2 protein expression in microvascular NG2 (+), PDGFRβ(+) pericytes (Coate et al., 2020; Fignani et al., 2020). On the other hand, Steenblock et al. (2021) detected ACE2 protein in VCAM-1 (+) vascular cells only in one out of three healthy individuals.

Reports on the presence of SARS-CoV-2 receptors in the pancreas show conflicting results concerning their expression level in certain pancreatic cell types. There are multiple factors that might explain the disparities in the receptor expression between studies, including technical, methodological, and biological reasons. Various technical differences might arise, starting with the material studied, including fixed autopsy tissues, human primary islets, and hPSC-derived pancreatic cells. Tissues from deceased individuals might show the alterations attributable to the cause of death or the prior diseases. The hPSC-derived cells are a well-established platform for transcriptomic studies, yet their gene expression is not identical to primary cells. Human primary islets, while the closest to the native pancreatic cells, are also subject to harsh isolation methods. For instance, Yang et al. (2020) identified ACE2 protein expression in dispersed human primary islet cells, yet Coate et al. (2020) did not find ACE2 in intact islets, suggesting that islet cells might alter the ACE2 expression in response to dispersion. Aside from isolation and culture methods, also other processing procedures, including fixation, and staining conditions might influence the results. For example, Coate et al. (2020) used four commercially available anti-ACE2 antibodies, including antibodies used by Yang et al. (2020) and Fignani et al. (2020). Coate et al. (2020) consistently detected a similar staining pattern with all anti-ACE2 antibodies, which was however different from the ones obtained by Yang et al. (2020) and Fignani et al. (2020). Further, the different antibodies might give various staining patterns based on the recognition of different epitopes. Fignani et al. (2020) reported that while all three anti-ACE2 antibodies detected ACE2 in the microvasculature, only two showed ACE2 expression in pancreatic islets. The remaining antibody recognized ACE2 N-terminus that is missing in the short ACE2 isoform. This suggests that the short ACE2 isoform might be preferentially expressed in islets and could explain the discrepancies between stainings. Additionally, methods have different detection limits, which is particularly important when the expression is rare or weak.

Besides technical factors, differences in the expression of the SARS-CoV-2-associated proteins might reflect natural variability between the individuals. For example, ACE2 expression was reported to differ depending on subject age (Schurink et al., 2022) and sex (Fernández-Atucha et al., 2017). Further, health status like ongoing inflammation might alter ACE2 expression level (Kimura et al., 2020), (Suárez-Fariñas et al., 2021). Additionally, in T2D the mRNA expression of ACE2, TMPRSS2, and CTSL is upregulated in the majority of pancreatic cell types, with the exception of ACE2 being downregulated in α-, β-, and δ-cells (Kusmartseva, et al., 2020). Also, COVID-19 itself was shown to modulate ACE2 receptor expression (Wu C.-T. et al., 2021), which might either potentiate or diminish the infection. It remains an open question what other factors might be responsible for the variability of the expression of ACE2 and other SARS-CoV-2 entry receptors.

Interestingly, ACE2-related proteases, including TMPRSS2, and CTSL (Kusmartseva, et al., 2020) show much broader expression than ACE2. For instance, ACE2, TMPRSS2, and CTSL mRNAs are expressed in 4.1, 53.7, and 20.7% of all acinar cells, respectively (Kusmartseva, et al., 2020). Yet, there is no evidence that TMPRSS2 and CTSL might enable SARS-CoV-2 entry without cooperation with ACE2, suggesting that ACE2 expression might be a limiting factor. The coexpression of ACE2 and TMPRSS2 is found only in 3.8%, and coexpression of ACE2 and CTSL in 1.5% of acinar cells (Kusmartseva, et al., 2020). Therefore, the assessment of the colocalization would be crucial to evaluate cell permissiveness to the virus.

While the expression is not as robust as in the respiratory tract, all pancreatic cell types express SARS-CoV-2 receptors. Varying combinations of SARS-CoV-2 receptors in pancreatic cell types might reflect different entry mechanisms, which consequently might translate into a need for different treatment options.

## Are Pancreatic Cells Infected by SARS-CoV-2?

In the previous chapter, we showed that different SARS-CoV-2 receptors are expressed in the pancreas, yet the level of expression and the fraction of receptor-expressing cells within the cell types remain a matter of debate. While the presence of receptor proteins suggests that cells might potentially be infected, it is not equivalent to successful infection. This chapter reviews the SARS-CoV-2 presence in the pancreatic cells (Figure 1). Based on the reports that quantified the percentage of SARS-CoV-2-infected cells, the highest rate of infection was demonstrated for β-cells, with on average 25% infected cells.

### β-cells

SARS-CoV-2 viral mRNA was detected at low levels by *in situ* hybridization in specimens from COVID-19 patients (Steenblock et al., 2021; Wu C.-T. et al., 2021). In human primary islets infected with SARS-CoV-2, SARS-CoV-2 transcripts were highly expressed in the subset of β-cells, as assayed by the scRNA-seq (Tang et al., 2021). Viral particles were detected by electron microscopy in cells containing insulin granules in tissues from SARS-CoV-2 (+) donors (Steenblock et al., 2021). Multiple studies found SARS-CoV-2 proteins in the β-cells in tissues from COVID-19 patients (Qadir et al., 2021; Shaharuddin et al., 2021; Tang et al., 2021; Wu C.-T. et al., 2021). Shaharuddin et al. (2021) found the SARS-CoV-2 S-protein in a subset of C-PEP (+) cells in COVID-19 donors. In the study by Fadh Qadir et al. (Qadir et al., 2021) N-protein colocalized with INS, yet only in one out of five SARS-CoV-2 (+) patients. Further, Wu C.-T. et al. (2021) demonstrated the presence of INS(+)/N-protein (+) cells in four out of seven examined COVID-19 subjects. Tang et al. (2021) demonstrated viral N-protein expression in varying fractions of INS(+) β-cells, from 7.7 to 15.5% of β-cells, in samples from five SARS-CoV-2 (+) individuals. In human primary islets infected *ex vivo* with SARS-CoV-2, Yang et al. (2020) identified the expression of S-protein in β-cells. 11.6% of INS(+)/N-protein (+) cells were found within infected human primary islets as assayed by flow cytometry (van der Heide et al., 2022). Of note, detailed mass cytometry analysis showed that N-protein and/or S-protein are coexpressed by 4% of β-cells and up to 48% of β-like cells, which are defined by high and lower INS expression, respectively (van der Heide). Further, Wu C.-T. et al. (2021) showed that ∼20% of human primary β-cells are permissive to SARS-CoV-2 infection, while in the study by Tang et al. (2021) the fraction of infected β-cells reached 40%. Intriguingly, Müller et al. (2021) found only a small fraction of C-PEP (+) cells coexpressing SARS-CoV-2 proteins, yet showed that 21% of NKX6.1 (+) cells were infected. In hPSC-derived β-cells infection with both SARS-CoV-2 pseudovirus and wildtype virus was confirmed. When hPSC-derived endocrine cells were transplanted into immunocompromised mice, which were further infected with SARS-CoV-2 pseudovirus, the virus was found in ∼60% of all β-cells (Yang et al., 2020), suggesting that β-cells are infected *in vivo* and that the degree of the infection might be higher than the one expected based on the *in vitro* studies.

### α-cells

High expression of viral mRNA in α-cells has been confirmed by scRNA-seq of the human primary islets infected with SARS-CoV-2 *ex vivo* (Tang et al., 2021). As for the protein, varying levels of α-cell infection were reported. Wu C.-T. et al. (2021) showed that 3–5% of α-cells from primary islets incubated with SARS-CoV-2 also expressed viral N-protein and/or S-protein. Further, in the study by van der Heide et al. (2022), 2.6% of GCG (+)/N-protein (+) cells were detected by flow cytometry in the samples from seven donors. Simultaneously, detailed proteomic mass cytometry analysis showed infection in 1% of α-cells and 1.8% of α-like cells, which show phenotypic similarity to α-cells but with lower GCG expression (van der Heide et al., 2022). Furthermore, Yang et al. (2020) demonstrated that α-cells are permissive for both pseudo- and wild-type SARS-CoV-2. In contrast, Müller et al. (2021) found that GCG (+) cells in human primary islets infected with SARS-CoV-2 were not expressing neither S-protein nor N-protein. Yang et al. (2020) detected SARS-CoV-2 pseudovirus also in hPSC-derived α-cells. Additionally, when hPCS-derived endocrine cells were transplanted into immunocompromised SCID-beige mice, which were further inoculated with SARS-CoV-2 pseudovirus, the percentage of infected α-cells reached ∼55%, proving α-cell permissiveness *in vivo* (Yang et al., 2020).

### δ-cells

ScRNA-seq performed on the human primary islets incubated with SARS-CoV-2 showed low expression of viral RNA in δ-cells (Tang et al., 2021). At the protein level, 2–10% of δ-cells were positively stained for viral N-protein and/or S-protein in the human primary islets infected with SARS-CoV-2 *ex vivo* (Wu C.-T. et al., 2021) Conversely, in the study by Müller et al. (2021), δ-cells were not permissive to the SARS-CoV-2 infection. Similarly, Yang et al. (2020) found that hPSC-derived δ-cells were not infected by SARS-CoV-2 pseudovirus.

### Acinar Cells

SARS-CoV-2 viral RNA was highly expressed in acinar cells, as shown by the scRNA-seq of the pancreatic tissues infected with SARS-CoV-2 *ex vivo* (Tang et al., 2021). Also, hPSC-derived acinar cells infected with SARS-CoV-2 showed by RT-qPCR the expression of viral mRNA (Shaharuddin et al., 2021). Immunostaining in the pancreas from five COVID-19 patients showed that 0.8–15.4% of Trypsin (+) acinar cells expressed SARS-CoV-2 N-protein (Tang et al., 2021). SARS-CoV-2 S-protein was found in the majority of CTRC (+) acinar cells and in a subset of AMY(+) acinar cells in fixed tissues from SARS-CoV-2 (+) individuals (Shaharuddin et al., 2021). Yang et al. (2020) infected primary pancreatic tissue with SARS-CoV-2, to reveal that 5.1% of AAT (+) acinar cells are costained for viral N-protein. In the study by van der Heide (van der Heide et al., 2022), only 0.6% of the acinar cells coexpressed N-protein and/or S-protein. In hPSC-derived acinar cells, up to 1% were infected when incubated with SARS-CoV-2 (Shaharuddin et al., 2021).

### Duct Cells

Duct cells showed a high expression of SARS-CoV-2 viral mRNA in the scRNA-seq of the pancreatic tissue (Tang et al., 2021). The RT-qPCR analysis detected viral mRNA in 20% of hPSC-derived ducts (Shaharuddin et al., 2021). The electron microscopy of the pancreas revealed viral particles in the ducts of only one out of five COVID-19 patients (Qadir et al., 2021). SARS-CoV-2 N-protein was observed in ducts cells in several studies (Kusmartseva, et al., 2020; Qadir et al., 2021; Tang et al., 2021). In tissue samples from two out of five COVID-19 patients, on average 2% of KRT19(+) ducts expressed N-protein (Qadir et al., 2021). Similarly, in the study by Tang et al. (2021) N-protein was found in 1.9–9.0% of KRT19(+) ducts across the samples from five SARS-CoV-2 (+) individuals. Contrary to that, Shaharuddin et al. (2021) did not detect SARS-CoV-2 protein in ducts, yet found that it is expressed in ∼18% of hPSC-derived ducts incubated with SARS-CoV-2. As for human primary islets infected *ex vivo* with SARS-CoV-2, van der Heide et al. (2022) reported that 1.1% of KRT19(+) cells were also positive for N-protein and/or S-protein as assayed by mass cytometry, while Tang et al. (2021) did not find any N-protein (+) duct cells.

### Pancreatic Microvasculature

At the mRNA level, SARS-CoV-2 N-protein transcript was detected in pancreatic endothelial cells of COVID-19 subjects, as assayed by scRNA-seq (Tang et al., 2021). Electron microscopy revealed SARS-CoV-2 particles in the microvasculature of one out of five COVID-19 individuals. At the protein level, N-protein was observed by Steenblock et al. (2021) in the endothelium by cellular fluorescence density measurement. By immunostaining the tissues from SARS-CoV-2 patients, Fadh Qadir et al. (Qadir et al., 2021) demonstrated 0.8% of CD31 (+)/N-protein (+) endothelial cells. Further, in the study by Tang et al. (2021), CD31 (+)/N-protein (+) cells accounted for 2.3–21% of total endothelial cells, depending on the patient. Human primary islets were also shown to be permissible for SARS-CoV-2 infection, with from 1.1% (van der Heide et al., 2022), up to 5% (Wu C.-T. et al., 2021) of CD31 (+) cells positive for N-protein and/or S-protein. Additionally, van der Heide et al. (2022) detected SARS-CoV-2 proteins in 22% of cells expressing CD34, another endothelial marker, and in 27% of pericytes.

## Impact of SARS-CoV-2 Infection on Pancreatic Cells

Among the consequences of SARS-CoV-2 infection in the human pancreas, several scenarios are emerging, including cell death, transdifferentiation or dedifferentiation, changes in morphology, tissue damage, inflammation, and loss of β-cell function. This indicates a deleterious effect of SARS-CoV-2 infection on pancreatic function.

van der Heide et al. (2022) reported that in human pancreatic islets infected with SARS-CoV-2 *ex vivo*, cell death in SARS-CoV-2 (+) cells is only slightly increased. Conversely, in the TUNEL assay performed by Wu C.-T. et al. (2021) in primary human islets, SARS-CoV-2 infection increased the number of apoptotic cells almost 2-fold for α-cells and ∼3-fold for β-cells. Interestingly, the treatment with just SARS-CoV-2 S-protein was sufficient to trigger β-cell apoptosis, with a ∼6-fold increase in TUNEL (+)/INS(+) cells (Wu C.-T. et al., 2021). Analysis of the phosphoproteome and quantification of kinase activity revealed upregulation of MAPK signaling, including JNK/p38 and PAK, two classical pro-apoptotic pathways in human primary islets incubated with SARS-CoV-2 or S-protein (Wu C.-T. et al., 2021). Further, in hPSC-derived endocrine cells inoculated with SARS-CoV-2, the expression of an apoptotic marker, cleaved CASP3, increased ∼80-fold for α-cells and ∼10-fold for β-cells (Yang et al., 2020). RNA-seq and subsequent gene set enrichment analyses (GSEA) from two independent groups showed the upregulation of apoptotic pathway in both human primary islets (Müller et al., 2021) and hPCS-derived endocrine cells (Yang et al., 2020) in response to SARS-CoV-2 infection. Additionally, upon infection with SARS-CoV-2, hPSC-derived ducts show an abnormal cytoplasmic localization of KRT19, become enlarged, and form multinucleated syncytia, the latter of which is known to amplify pro-apoptotic signals (Shaharuddin et al., 2021). Of note, one report examined another mechanism of cell death, necroptosis. Immunostaining against phosphorylated MLKL, a necroptotic marker, showed a significant increase in the necroptosis among both endocrine and exocrine cells in tissues from 11 COVID-19 patients (Steenblock et al., 2021).

Several studies suggested that pancreatic endocrine cells dedifferentiate or transdifferentiate upon SARS-CoV-2 infection. Transcriptomic profiling performed showed downregulation in genes associated with α- and β-cell physiology, including glucagon and calcium signaling pathways (Yang et al., 2020; Müller et al., 2021). ScRNA-seq of human primary islets infected with SARS-CoV-2 revealed decreased INS expression and upregulation of α-cell and acinar cell markers (Tang et al., 2021). Additionally, Shaharuddin et al. (2021) showed by RT-qPCR that the expression of ductal markers, KRT19, CFTR, CA2, and HNF1β is also increased in COVID-19 individuals. At the protein level, immunostaining in tissue samples from five COVID-19 patients and in SARS-CoV-2-infected human primary islets, confirmed an increased percentage of GCG (+)/INS(+) and Trypsin1 (+)/INS(+) cells, with lower INS and higher GCG and Trypsin1 (acinar cell marker) fluorescence intensity in β-cells (Tang et al., 2021). SARS-CoV-2 infection of human primary islets. As for the possible mechanism of transdifferentiation, based on the scRNA-seq of SARS-CoV-2-infected human primary cells, Tang et al. (2021) suggested activation of cellular stress response via eIF2α phosphorylation, which was confirmed by increased phosphorylation of PKR and eIF2α proteins in infected EndoC-βH1 cells. Elevated cellular stress was further validated by staining against G3BP1, a component of stress granules, in SARS-CoV-2-infected human primary cells, which revealed an increase in both the number and average intensity of stress granules (Tang et al., 2021). In contrast, van der Heide et al. (2022) found no differences in cell proportions between SARS-CoV-2 infected and control samples and no changes in α- and acinar cell markers in infected β-cells, contradicting transdifferentiation of endocrine cells upon SARS-Cov-2 infection. Instead, they proposed that SARS-CoV-2 might utilize host transcriptional machinery leading to the increased viral and decreased host protein production, as SARS-CoV-2 N-protein load was inversely related to hormone expression (van der Heide et al., 2022). Müller et al. (2021)) found that in SARS-CoV-2 infected primary endocrine cells, the vast majority of N/S-protein (+) cells were hormone (-). However, many N/S-protein (+) cells expressed NKX6.1, a transcription factor that beyond pancreatic fetal development is only sustained in β-cells (Müller et al., 2021). This suggests that INS(+) β-cells lost INS expression upon SARS-CoV-2 infection, potentially indicating β-cell dedifferentiation. Also, in tissues from COVID-19 individuals, a subset of infected cells did not morphologically resemble normal ductal, acinar, or endocrine cells (Müller et al., 2021).

Pancreatic tissue damage was observed in five COVID-19 patients, including multiple blood clots in pancreatic vascularization and a 2-fold increase in fibrotic tissue area (Qadir et al., 2021). In human primary islets infected with SARS-CoV-2, Müller et al. (2021) noted dilated and excessively vacuolated endoplasmic reticulum-Golgi apparatus complex, which is an indicator of endoplasmic reticulum stress.

Several studies analyzed the functionality of SARS-CoV-2 infected human primary β-cells. Wu C.-T. et al. (2021) and van der Heide et al. (2022) both reported a small, yet significant decrease in total INS secretion. Further, static glucose-stimulated insulin secretion (GSIS) was also disturbed upon SARS-CoV-2 infection, with ok 1.5-fold (Wu C.-T. et al., 2021) or even 4-fold (Müller et al., 2021) reduction in fold insulin induction. Despite similar trends, Tang et al. (2021) did not detect a significant reduction either in total INS secreted in response to KCl stimulation or in GSIS, possibly due to the high variability.

Multiple studies reported upregulation in the inflammation and stress response pathways, and chemokine expression. Transcriptomic profiling of SARS-CoV-2-infected primary islets showed an upregulation in pathways associated with viral infections, defense response to virus, regulation of viral replication, cellular stress response, eIF2 signaling, interferon (IFN) signaling (Yang et al., 2020; Müller et al., 2021; Tang et al., 2021; van der Heide et al., 2022). Detailed analysis of human primary islets infected with SARS-CoV-2 showed the upregulation of chemokine expression, including CCL2, CXCL5, and CXCL6 (Yang et al., 2020). Similarly, scRNA-seq detected a robust upregulation in the expression of numerous chemokines and cytokines, such as CCL2, CXCL2, CXCL1, CCL4, CCL3, CXCL5, CCL8, IL1RN, and IL1B, which was further confirmed by ELISA (Tang et al., 2021)). Similar findings were reported by Shaharuddin et al. (2021) for hPSC-derived duct and acinar cells infected with SARS-CoV-2, where transcriptomic profiling showed upregulation in chemokines and immunoregulatory genes, such as CXCL12, CXCL6, CCR7, PTGES, MIF, and STAT3. In contrast, van der Heide et al. (2022) assessed levels of inflammation-associated proteins in the culture medium after infection of primary islets with SARS-CoV-2 and detected only slightly elevated levels of chemokines CXCL10 and CXCL11, and their inducer, INFγ. Further, immune cell infiltration of the pancreas was assessed in tissues from 11 COVID-19 patients, showing an increased number of CD45 (+) cells in both endocrine and exocrine compartments (Steenblock et al., 2021). Additionally, an almost 5-fold increase in the expression of the endothelial inflammation marker protein, ICAM1, was reported for SARS-CoV-2 (+) subjects (Qadir et al., 2021).

## Summary

SARS-CoV-2 infection and diabetes are involved in a bidirectional relationship. Diabetes is one of the leading comorbidities increasing COVID-19 severity, ICU admission rates, and mortality. Moreover, infection with SARS-CoV-2 can precipitate pancreatitis and new-onset diabetes, also in previously healthy individuals. We reviewed the expression of the canonical and alternative SARS-CoV-2 receptors and the permissiveness to the SARS-CoV-2 infection in different types of pancreatic cells. While receptor expression in pancreatic cells is not as robust as in the respiratory tract, pancreatic cells become infected. Collectively, multiple studies imply that SARS-CoV-2 infection impairs pancreatic cell survival and function. However, it requires more longitudinal clinical and molecular studies to better understand the molecular changes caused by SARS-CoV-2 in β-cells and whether they underlie the development of diabetes.
